# CenTauR: Toward a universal scale and masks for standardizing tau imaging studies

**DOI:** 10.1002/dad2.12454

**Published:** 2023-07-07

**Authors:** Victor L. Villemagne, Antoine Leuzy, Sandra Sanabria Bohorquez, Santiago Bullich, Hitoshi Shimada, Christopher C. Rowe, Pierrick Bourgeat, Brian Lopresti, Kun Huang, Natasha Krishnadas, Jurgen Fripp, Yuhei Takado, Alexandra Gogola, Davneet Minhas, Robby Weimer, Makoto Higuchi, Andrew Stephens, Oskar Hansson, Vincent Doré, Alzheimer's Disease Neuroimaging Initiative and the AIBL research group

**Affiliations:** ^1^ Department of Psychiatry University of Pittsburgh Pittsburgh Pennsylvania USA; ^2^ Department of Molecular Imaging & Therapy Austin Health Melbourne Victoria Australia; ^3^ Clinical Memory Research Unit Department of Clinical Sciences Lund University Malmö Sweden; ^4^ Genentech Inc. South San Francisco California USA; ^5^ Life Molecular Imaging GmbH Berlin Germany; ^6^ Department of Functional Brain Imaging National Institutes for Quantum and Radiological Science and Technology Chiba Japan; ^7^ Brain Research Institute Niigata University Niigata Japan; ^8^ Florey Department of Neurosciences & Mental Health The University of Melbourne Melbourne Parkville Australia; ^9^ The Australian Dementia Network (ADNeT) Melbourne Victoria Australia; ^10^ Health and Biosecurity Flagship The Australian eHealth Research Centre CSIRO Brisbane Queensland Australia; ^11^ Department of Radiology University of Pittsburgh Pittsburgh Pennsylvania USA; ^12^ Florey Institute of Neurosciences & Mental Health Parkville Victoria Australia; ^13^ Memory Clinic Skåne University Hospital Malmö Sweden; ^14^ Health and Biosecurity Flagship The Australian eHealth Research Centre CSIRO Heidelberg Victoria Australia

## Abstract

**INTRODUCTION:**

Recently, an increasing number of tau tracers have become available. There is a need to standardize quantitative tau measures across tracers, supporting a universal scale. We developed several cortical tau masks and applied them to generate a tau imaging universal scale.

**METHOD:**

One thousand forty‐five participants underwent tau scans with either ^18^F‐flortaucipir, ^18^F‐MK6240, ^18^F‐PI2620, ^18^F‐PM‐PBB3, ^18^F‐GTP1, or ^18^F‐RO948. The universal mask was generated from cognitively unimpaired amyloid beta (Aβ)− subjects and Alzheimer's disease (AD) patients with Aβ+. Four additional regional cortical masks were defined within the constraints of the universal mask. A universal scale, the CenTauR_z_, was constructed.

**RESULTS:**

None of the regions known to display off‐target signal were included in the masks. The CenTauR_z_ allows robust discrimination between low and high levels of tau deposits.

**DISCUSSION:**

We constructed several tau‐specific cortical masks for the AD continuum and a universal standard scale designed to capture the location and degree of abnormality that can be applied across tracers and across centers. The masks are freely available at https://www.gaain.org/centaur‐project.

## 1 BACKGROUND

Tau positron emission tomography (PET) imaging is the most recent addition to the arsenal of tools for the in vivo assessment of neurodegenerative proteinopathies. Prior to this development, the presence and extent of aggregated tau in the brain could only be characterized using *post mortem* examination.^1^ Despite the challenges inherent to imaging tau pathology, which include its intracellular location; the presence of multiple human tau isoforms (three repeat [3R], four repeat [4R]) and morphologies (paired helical filament [PHF], straight filament [SF]); numerous post‐translational modifications (e.g., phosphorylation, truncation, nitration); and, in the case of Alzheimer's disease (AD), lower concentrations than amyloid beta (Αβ) in colocalizing tau and Aβ deposits (for review see Villemagne et al.^2^ and Mathis et al.^3^), there has been a tremendous amount of progress in the last few years, with several selective tau tracers identified and increasingly used for human imaging studies. These tracers have been shown to be largely specific for the mixed 3R/4R PHF tau pathology characteristic of AD and Down syndrome and have helped further our understanding of tauopathies as well as the relationships among Aβ, tau, neurodegeneration, and cognitive decline in AD.^4^, ^5^, ^6^, ^7^, ^8^, ^9^, ^10^, ^11^

In addition to the idiosyncratic characteristics of tau aggregates, and their asymmetric and heterogeneous brain distribution, a major obstacle to the widespread implementation of tau imaging in therapeutic trials or comparing the findings of investigational imaging studies across cohorts and institutions is that tau tracers differ in their molecular structures and display a range of tau binding affinities, in vivo kinetics, and degree of non‐specific binding, as well as distinct regional patterns of “off‐target” and non‐specific binding. Such differences lead to disparities in PET‐derived standardized uptake value ratio (SUVR) measurements between tracers, as highlighted by several head‐to‐head studies comparing different tau tracers.^12^, ^13^ It is also important to note that most of these tau tracers do not reach apparent steady state in regions with high tau pathology during the scanning period, and while the use of semi‐quantitative estimates such as SUVR was adopted early in the implementation of these tracers as a compromise to make PET imaging studies less burdensome to clinical populations, a priori kinetic modeling studies of tau tracers in early development stages may have led to further optimization of scanning protocols to be less biased to tau signal.^14^, ^15^, ^16^, ^17^ When added to the use of diverse quantitative approaches and different regions of interest, these methodological differences conspire to decrease reproducibility and pose a challenge when trying to compare tau outcomes across cohorts or in therapeutic trials that use different tau tracers. A further obstacle within the tau PET field is the definition of a reliable, consistent, and reproducible threshold of abnormality across tracers. One of the issues relates to the actual utility of a cut‐off given the continuous nature of Aβ or tau deposition.^18^, ^19^ While thresholds are arbitrary, to adopt one, it needs to be shown that it is relevant and accurate from a diagnostic and/or prognostic point of view.^20^, ^21^ In essence, biomarker thresholds should be adopted for a specific purpose that is directly related to the clinical question under scrutiny. From a clinical perspective, a visual binary (positive/negative) status will help separate those subjects with a significant aggregated protein burden in the brain that is likely to explain the clinical syndrome from those with a low pathologic burden that is likely to be clinically insignificant. Similar dilemmas arise in research settings.

In response to similar challenges faced earlier with Aβ PET,^22^ a standardization method was developed whereby Aβ PET outcome data acquired using different Aβ tracers and methods was normalized to a 100‐point scale, the units of which were termed “Centiloids,” using a linear scaling procedure.^22^ While the method transforms all Aβ tracers’ semiquantitative results into a single universal scale and because sampling was only based on ^11^C‐Pittsburgh compound B, the idiosyncratic binding properties of these Aβ tracers remain unaccounted for so they might be more or less sensitive or accurate for making a statement about a similar index of cerebral Aβ burden. Furthermore, while the pattern of Aβ deposition throughout the brain is relatively uniform across subjects, and thus a single universal target mask provides reproducible statements of Aβ in the brain, the deposition of tau, especially at the early stages, tends to be more heterogeneous,^23^ requiring a more regional approach to the sampling of target areas.

In the present study, we aimed to standardize tau PET results by establishing the location and amount of abnormality of tau aggregates in the brain, and expressing them in a universal standard scale, the unit of which are termed “CenTauR”—using tau PET data from the six most commonly used tracers (^18^F‐flortaucipir, ^18^F‐MK6240, ^18^F‐PI2620, ^18^F‐PM‐PBB3, ^18^F‐RO948, and ^18^F‐GTP1) and an approach similar to the one used in the Centiloid project.

## 2 METHODS

This study involved 1045 participants from various cohorts (Australian Imaging Biomarkers and Lifestyle study [AIBL], Alzheimer's Disease Neuroimaging Initiative [ADNI], Biomarkers for Identifying Neurodegenerative Disorders Early and Reliably [BioFINDER]), academic institutions (National Institutes for Quantum and Radiological Science and Technology, Chiba), as well as industry (Life Molecular Imaging, Genentech). All participants underwent a tau PET scan (300 MK6240, 503 FTP, 47 PI2620, 57 R0948, 87 GTP1, 51 PM‐PBB3) and a structural magnetic resonance imaging (MRI; for complete details, see Method S1 in supporting information). All participants were assigned a diagnosis of cognitively unimpaired (CU), mild cognitive impairment (MCI), or AD dementia or other dementia (OD) by the entity providing the data. Criteria for assigning participant diagnosis can be found elsewhere.^15^, ^24^, ^25^, ^26^, ^27^ Aβ status (Aβ+ or Aβ−) was defined using either Aβ PET or the Aβ42/Aβ40 ratio in cerebrospinal fluid (CSF). Analysis of variance was used to determine any significant demographic difference between cohorts.

### 2.1 Image processing

Tau scans were spatially normalized using principal component analysis (PCA) based on Computational Analysis of PET by AIBL (CapAIBL),^28^ which is a publicly available cloud‐based platform in which PET images are spatially normalized to a standard template using an adaptive atlas approach (https://capaibl‐milxcloud.csiro.au), and Statistical Parametric Mapping (SPM, version 8) using the standard pipeline for the Centiloid method (CL‐SPM) described in Klunk et al.^22^ For more detailed information on the Centiloid pipeline, including MATLAB commands, please refer to Method S2 in the supporting information. All spatially normalized scans were visually assessed to ensure proper registration, especially in the mesial temporal lobe (MTL).^29^ In the case of CL‐SPM, all scans that did not pass visual assessment were reprocessed using a different orientation matrix until they passed a visual quality check (QC). Scans that failed visual QC three times in a row were excluded from further analysis. In the CU group, Aβ− scans were excluded if the presence of tau was visually detected in the cortex or in the MTL. We defined a sub‐cerebellar cortex region based on the Centiloid cerebellum cortex mask as reference region, excluding the upper portion (slice > −37) of the cerebellum to avoid off‐target binding often observed in the cerebellar vermis, and also the lower part (slice < −47) to avoid quantification challenges such as partial volume, low axial sensitivity, and out‐of‐field scatter (Figure S1 in supporting information). The same reference region was used for the CL‐SPM and CapAIBL pipelines.

**RESEARCH IN CONTEXT**

1. **Systematic review**: The authors reviewed the literature using traditional (e.g., PubMed) sources and meeting abstracts and presentations. While the use of tau positron emission tomography (PET) imaging rapidly increased in research and in clinical trials over the past few years, there is no standardization pipeline for the quantification of tau imaging across tau tracers and quantification software.
2. **Interpretation**: We built a global and several regional universal masks for the sampling of tau PET scans based on the most commonly used tau PET tracers. We then derived a universal scale across tracers, the CenTauR_z_, to measure the tau signal.
3. **Future directions**: Standardized quantification will facilitate the derivation of universal cut‐off values, merging of large cohorts, and comparison of longitudinal changes across tracers and cohorts both in clinical studies and therapeutic trials.

For each tracer and normalization approach (i.e., CapAIBL, CL‐SPM), we averaged all CU Aβ− and AD Aβ+ scans separately, generating mean CU Aβ− and AD Aβ+ images. We then subtracted the CU Aβ− mean image from the AD Aβ+ mean image to generate a difference image. After exploring several thresholds, the resultant difference‐image was thresholded at one third of the difference in the inferior temporal lobe. This threshold produced large and consistent volumes of interest across tracers of areas of the brain with the greatest tau load. We then constructed a “universal” tau mask from the intersection (i.e., spatial overlap) of the six tracer‐specific masks. An MRI‐derived gray matter mask obtained from the FreeSurfer segmentation of 100 MRIs (independent dataset) at PET resolution was then applied to the universal mask to only sample cortical regions. The resulting mask was then mirrored and fused to remove the hemispherical asymmetry of tau pathology. Last, an additional four subregions were defined within the constraints of the universal mask: Mesial Temporal, Meta Temporal, Temporo‐Parietal, and Frontal ROIs (regions of interest; Method S3 in supporting information). Agreement between masks was assessed using the Dice index, which is a measure of the similarity between various images. Finally, for each tracer, the mean and standard deviation of the CU Aβ− subjects were used to generate CenTauR *z* scores in each of the five ROIs, similar to what was previously proposed by Vemuri et al.^30^

### 2.2 Visual topographical subtype classification

Seventy‐eight ^18^F ‐MK6240 AD Aβ+ scans from the AIBL cohort were visually rated by two readers (C.C.R. and N.K.), blind to participant characteristics, resulting in consensus visual reads, as previously described.^31^ Briefly, scans were rated as (1) tau negative (no tracer retention or minimal [unilateral or bilateral]) entorhinal cortex retention, (2) limbic predominant (pronounced tracer retention in the MTL with no cortical retention), (iii) hippocampal sparing (cortical tau tracer retention with no or minimal MTL signal), or (4) typical (MTL and cortical tracer retention).

## 3 RESULTS

Participant characteristics by tau PET tracer are summarized in Table S1 in supporting information. Overall, participants from the ^18^F‐GTP1 and ^18^F‐PM‐PBB3 cohorts were significantly younger compared to participants from the other cohorts. Compared to the MCI and AD dementia groups, CU Aβ+ participants were significantly older (F‐stat = 3.9, *P* < 0.002) and had fewer males (F‐stat = 3, *P* < 0.005). No significant differences in age, sex, Mini‐Mental State Examination or Clinical Dementia Rating were found between the AD Aβ+ patients from the different cohorts (F‐stat = 1, *P* = 0.4).

### 3.1 Tau mask sampling

Twenty‐three scans (eight ^18^F‐RO948, one ^18^F‐GTP1, five ^18^F‐PI2620, one ^18^F‐FTP, eight ^18^F‐PM‐PBB3) did not pass visual QC using the CL‐SPM pipeline or did not have an MRI of sufficient quality while only one scan did not pass visual QC using both CapAIBL and CL‐SPM. A further six CU Aβ– were visually excluded due to the presence of tracer uptake in the MTL. These 29 scans were excluded from further analysis.

**FIGURE 1:**
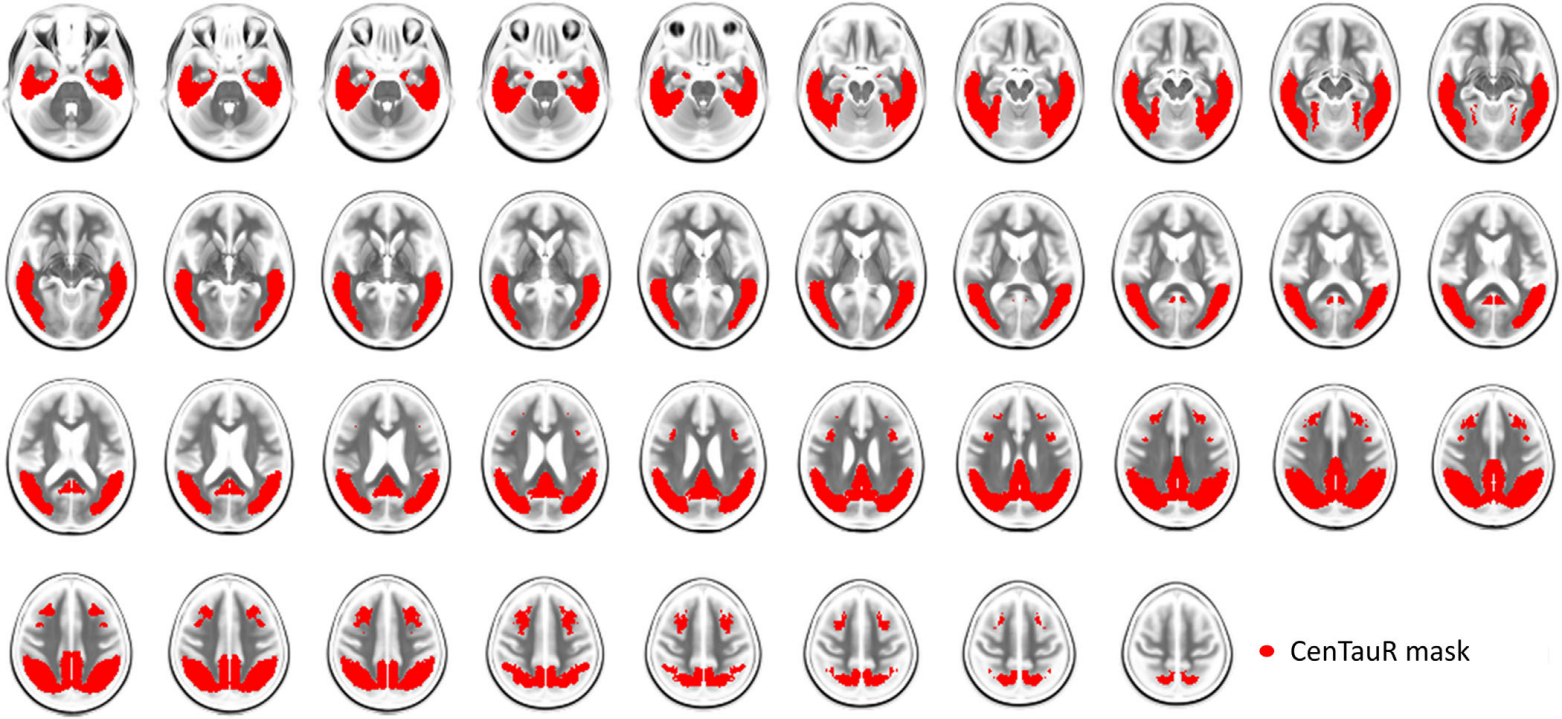
CenTauR mask overlaid on a magnetic resonance imaging template.

CL‐SPM tracer‐specific masks showed a reasonable overlap (Figure S2 in supporting information), with a global Dice score of 0.58 (95% confidence interval [CI], 0.52–0.61) and a Dice score in the cortical mask of 0.61 (95% CI, 0.60–0.69). The mean Dice score obtained when comparing paired tracer‐specific masks was 0.85 (Table S2 in supporting information). All masks included the mesial temporal, meta‐temporal, posterior cingulate/precuneus, and subfrontal regions. The CenTauR mask overlaid on an MRI template is shown in Figure 1, while the subregion masks are shown in Figure S3 in supporting information. None of the known off‐target signal regions were discernible in the five masks (Figure S4 in supporting information).

Both quantitative pipelines provided very similar tau masks, with a Dice score of 0.75 between universal masks generated using CapAIBL and CL‐SPM. Part of this difference was due to the normalized space of CapAIBL, which is different from the Montreal Neurological Institute space, the CL‐SPM mask required resampling to be compared to the CapAIBL mask. In the remainder of this paper, we only use the masks defined using the CL‐SPM pipeline.

### 3.2 CenTauR_z_ quantification

**FIGURE 2:**
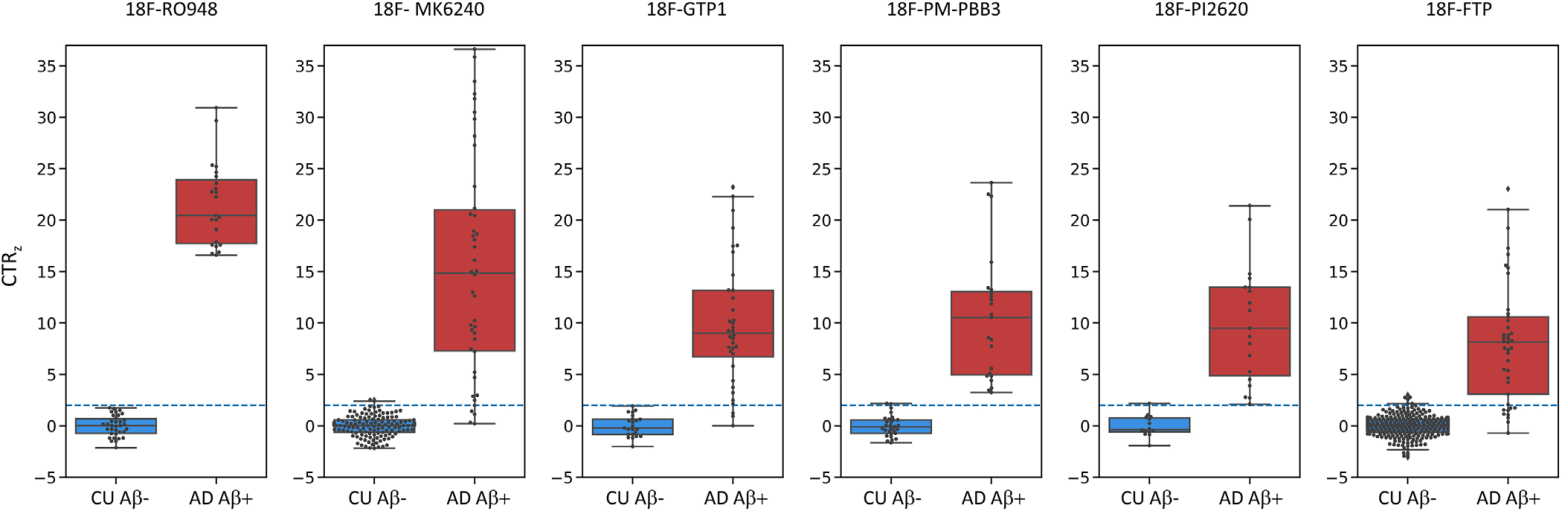
Comparisons of the CTR_z_ in the Meta Temporal ROI between CU Aβ– and AD Aβ+ for the six tau tracers. The blue dashed line corresponds to two CTR_z_. Aβ, amyloid beta; AD, Alzheimer's disease; Aβ, amyloid beta;  CTR_z_, CenTauR_z_; CU, cognitively unimpaired; ROI, region of interest.

Table 1 provides the regional equations to convert CL‐SPM–based SUVR values (Material S2 in supporting information) into CenTauR_z_ (CTR_z_) for each of the six tau tracers included in the study. Figure 2 displays the box plot of the Meta Temporal CTR_z_ for CU Aβ− and AD Aβ+ individuals. CTR_z_ for the other four ROIs are presented in Figure S5 in supporting information and CapAIBL CTR_z_ are displayed in Figure S6 in supporting information. Using a threshold of two CTR_z_ in the Meta Temporal ROI, all tracers showed high discriminative accuracy for the separation of AD Aβ+ from CU Aβ− individuals (accuracy = 0.96 [min = 0.95 − max = 1], sensitivity = 0.91 [0.78 − 1], specificity = 0.97 [0.93 − 1]) with mean CTR_z_ scores for the six different AD cohorts ranging from 8.1 to 22 (Figure 2 and Figure S7 in supporting information). Similar accuracies were observed using the Mesial Temporal (accuracy = 0.95 [0.90 − 1], sensitivity = 0.90 [0.83 − 1], specificity = 0.97 [0.95 − 1]) and Temporo‐Parietal (accuracy = 0.94 [0.90 − 1], sensitivity = 0.88 [0.76 − 1], specificity = 0.96 [0.95 − 1]) ROIs, while the accuracy for the Frontal ROI (accuracy = 0.91 [0.81 − 1]) was somewhat lower due to lower sensitivity (sensitivity = 0.73 [0.5 − 1]); whereas specificity (specificity = 0.97 [0.91, 1]) was similar to that for the Meta Temporal ROI.

**TABLE 1:** Conversion equations from Statistical Parametric Mapping standardized uptake value ratios to CenTauR_z_.

Tracer	Universal mask	Mesial temporal	Meta temporal	Temporo parietal	Frontal
^18^F‐RO948	13.05**x**–15.57	11.76**x**–13.08	13.16**x**–16.19	13.05**x**–15.62	12.61**x**–13.45
^18^F‐FTP	13.63**x**–15.85	10.42**x**–12.11	12.95**x**–15.37	13.75**x**–15.92	11.61**x**–13.01
^18^F‐MK6240	10.08**x**–10.06	7.28**x**–7.01	9.36**x**–10.6	9.98**x**–10.15	10.05**x**–8.91
^18^F‐GTP1	10.67**x**–11.92	7.88**x**–8.75	9.60**x**–11.10	10.84**x**–12.27	9.41**x**–9.71
^18^F‐PM‐PBB3	16.73**x**–15.34	7.97**x**–7.83	11.78**x**–11.21	16.16**x**–14.68	15.7**x**–13.18
^18^F‐PI2620	8.45**x**–9.61	6.03**x**–6.83	7.78**x**–9.33	8.21**x**–9.52	9.07**x**–9.01

**FIGURE 3:**
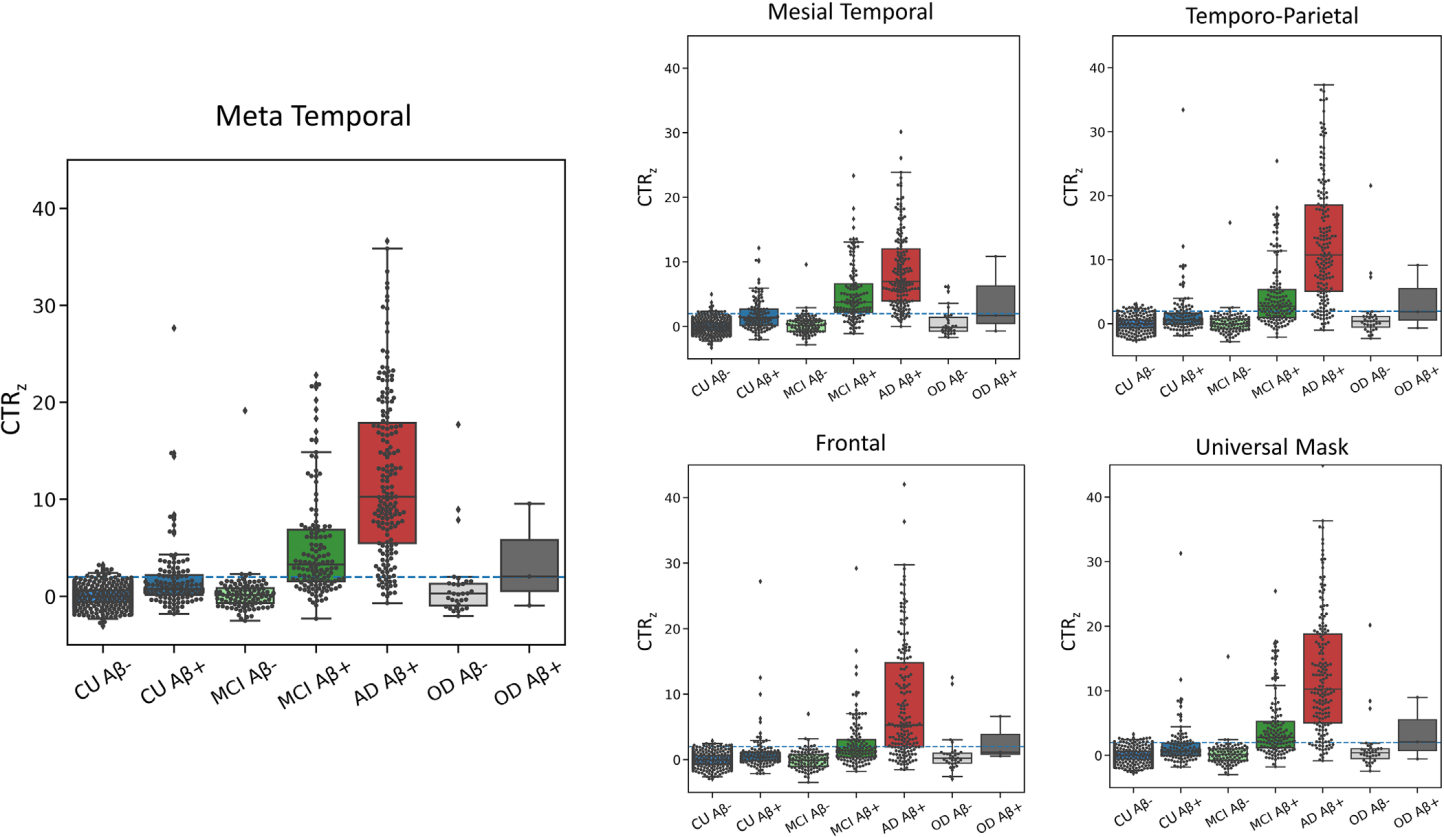
Boxplots of the ROI CTR_z_ of all participants from all cohorts in the different ROI. The blue dashed line corresponds to two CTR_z_. Aβ, amyloid beta; AD, Alzheimer's disease; CTR_z_, CenTauR_z;_ CU, cognitively unimpaired; MCI, mild cognitive impairment; OD, other dementia; ROI, region of interest.

Figure 3 and Figure S8 in supporting information show boxplots of CTR_z_ scores in the five different ROIs. The AD Aβ+ group had significantly higher CTR_z_ scores across ROIs compared to all other cognitive groups (Welch's *T* > 7.6). CU Aβ+ had significantly higher CTR_z_ compared to CU Aβ− and MCI Aβ− in all regions with the strongest effect size in the Mesial Temporal ROI (Welch's *T* > 6) and the lowest in the Frontal ROI (Welch's *T* ≈ 3). Among the CU Aβ+, 36% had a CTR_z_ higher than 2 in the Mesial Temporal, 29% in the Meta Temporal, 21% in the Temporo‐Parietal, 12% in the Frontal, and 23% in the universal mask, while these prevalences were, respectively, 77%, 63%, 58%, 41%, 60% for the MCI Aβ+ group, and 91%, 90%, 87%, 73%, and 88% for the AD Aβ+ and around 4% and 2.5% in all regions for the MCI Aβ− and CU Aβ−, respectively.

### 3.3 CapAIBL versus CL‐SPM pipeline

**FIGURE 4:**
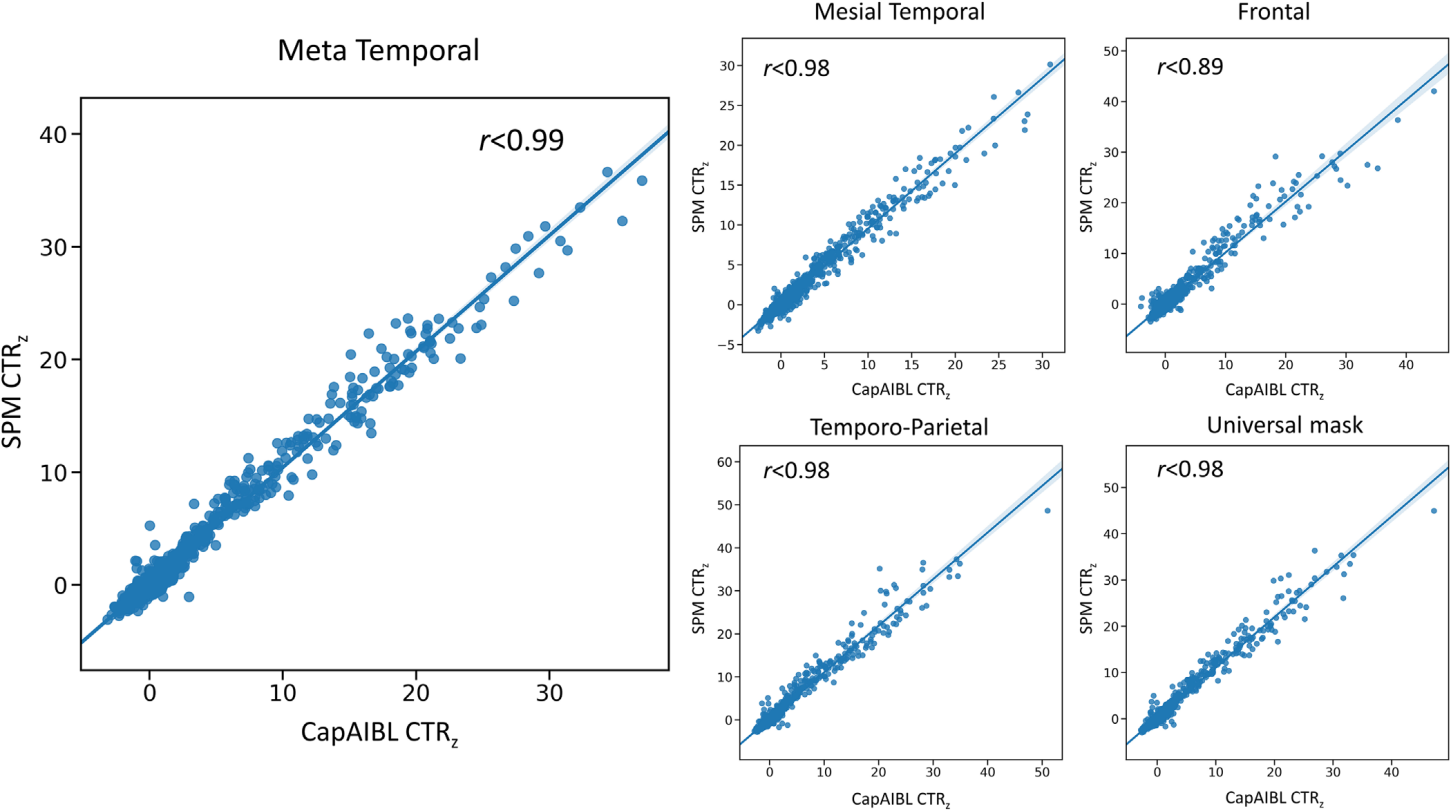
Comparison of the CTR_z_ generated with SPM (y‐axis) and with CapAIBL (x‐axis). CapAIBL, computational analysis of positron emission tomography by Australian Imaging, Biomarkers and Lifestyle study; CTR_z_, CenTauR_z_.

The equations to convert CapAIBL SUVR values into CTR_z_ scores are presented in Table S3 in supporting information. Converting slopes between CapAIBL and CL‐SPM were of the same rank order except for the Temporo‐Parietal and Frontal ROIs for ^18^F‐PM‐PBB3, due to the slightly higher standard deviation of the CapAIBL SUVRs in the CU Aβ− group. The correlation between CTR_z_ scores from CL‐SPM and CapAIBL was 0.99 in the Meta Temporal ROI; 0.98 in the Mesial, Temporo‐Parietal, and universal ROIs; and 0.89 in the Frontal ROI (Figure 4). Using an arbitrary threshold of 2.0 CapAIBL CTR_z_ in the Meta Temporal region, all tracers showed high discriminative accuracy for the separation of AD Aβ+ from CU Aβ− individuals (accuracy = 0.95 [0.93 − 1], sensitivity = 0.89 [0.78 − 1], specificity = 0.98 [0.96 − 1]), with mean CTR_z_ for the different AD cohorts ranging from 7.6 to 20.6 (Figures S6 and S9 in supporting information).

Figures S10 and S11 in supporting information display the association between CTR_z_ scores and Aβ+ PET Centiloid values across the five ROIs. As previously reported,^32^ individuals with a Centiloid value below 50 and a CTR_z_ value above 2 in the Meta Temporal or Temporo‐Parietal ROIs were rare but became increasingly more common as Centiloid values increased. Similarly, very few individuals with a Frontal CTR_z_ values above 2 had Centiloid values below 70. Scatter plots showing the relationship between CapAIBL‐derived CTR_z_ scores and Centiloids are shown in Figure S11.

**FIGURE 5:**
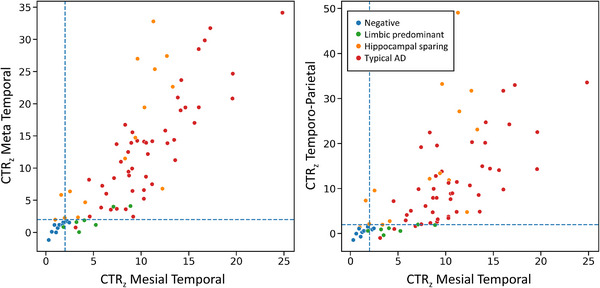
Scatter plots of the CTR_z_ in the Meta Temporal and Temporo Parietal as a function of the CTR_z_ in the Mesial Temporal from the ^18^F‐MK6240 AIBL cohort. Points are colored depending on their visual reads. The blue dashed lines correspond to two CTR_z_. AD, Alzheimer's disease; AIBL, Australian Imaging, Biomarkers and Lifestyle study; CTR_z_, CenTauR_z_.

Scatter plots showing CTR_z_ scores in the Meta Temporal and Temporo‐Parietal ROIs as a function of CTR_z_ scores in the Mesial Temporal for ^18^F‐MK6240 are presented in Figure 5. Visual classifications (i.e., tau negative, limbic predominant, hippocampal sparing, and typical) are color coded. A CTR_z_ > 2 in the Mesial Temporal ROI accurately differentiated tau negative scans from all other classifications (accuracy = 0.92, sensitivity = 0.97, specificity = 0.60); applying a threshold of 2 CTR_z_ in the Mesial Temporal and in the Meta Temporal together slightly increased the accuracy of detecting tau negative scans (accuracy = 0.94, sensitivity = 1.0, specificity = 0.60). Using CTR_z_ > 2 in Mesial Temporal ROI and <2 in the Meta Temporal ROI, yielded an accuracy of 0.92 to detect limbic‐predominant individuals. Using CapAIBL the specificities and accuracies were slightly improved (tau negative: accuracy = 0.95, sensitivity = 0.97, specificity = 0.80; limbic predominant: accuracy = 0.92, sensitivity = 0.96, specificity = 0.57, Figures S12–S13 in supporting information).

## 4 DISCUSSION

In the present work we described the CenTauR_z_ scale, a method that facilitates the expression of the level of abnormality of the semiquantitative tau PET signal at both a regional and global level. Also, the CenTauR_z_ scale allows, by incorporating the intrinsic “noise” of each tau tracer into the measurement, the generation of a universal scale of tau pathologic burden across tracers. The two pipelines used to quantify brain PET imaging (CapAIBL and CL‐SPM) generated consistent results in quantifying tau scans in all ROIs, with high discriminative power in distinguishing AD Aβ+ from CU Aβ− and tau negative scans from limbic predominant, hippocampal sparing, and typical AD tau scans when using a threshold of > 2 CTR_z_ in different ROIs.

An important aspect, both for clinical interpretation and for therapeutic trials, is the selection of brain regions sampled to capture the distribution of tau, how this index of tau load changes over time, and what CTR_z_ level is considered high tau.^33^ Given the low spatial resolution of PET, it can be counterproductive to impose a neuropathological piecemeal staging system, such as those proposed by Braak and Braak^34^ or Delacourte,^35^ to the sampling of tau PET images.^36^, ^37^ Atypical and heterogeneous presentations of tau deposits, and how they intimately relate to the clinical phenotype,^34^, ^35^ are missed by the incrementally sequential Braak staging. Applying the Braak or Delacourte staging^34^, ^35^ is further complicated by the different neuropathological subtypes of tau deposition in AD.^38^ From the pathological AD subtypes, only the typical (reported to be between 55%–75% in different series)^39^, ^40^, ^41^ completely fulfills the sequential Braak stages. Several reports have shown that a meta‐temporal region,^42^ or a temporoparietal (including posterior cingulate) AD‐signature region^43^, ^44^ outperforms the Braak staging for the early detection of cortical tau, for establishing the differential diagnosis of AD versus non‐AD neurodegenerative conditions,^45^ as well as for capturing longitudinal changes in cortical tau signal. These regions seem to perform reliably across different tau tracers and use sites and, despite these tracers presenting different dynamic ranges, they yielded the same cut‐off for abnormality in different cohorts.^46^ While the use of tau imaging for disease staging is strongly recommended,^47^ the use of neuropathological staging should be applied carefully, not as an a priori condition, but as the result of the actual observed pattern of tau deposition on the PET images. Furthermore, it has been shown that tau imaging, at least with ^18^F‐FTP,^48^ can reliably detect a B3 stage (equivalent to Braak V–VI), so attempting to classify earlier Braak stages using this tracer, with its high level of non‐specific binding,^49^ would likely yield less reliable results. Similar issues may apply to other tau tracers. Such considerations argue against using current neuropathological staging approaches, especially because it progresses from very small regions (Braak I–II) that are susceptible to partial volume effects and easily contaminated by off‐target binding, to very large regions (Braak V–VI) that encompass large portions of the cerebral cortex and subcortical structures, making it impractical for implementation in clinical studies, and foremost, in therapeutic trials. Our method is designed to capture tau levels and distribution in the brain as well as tau progression and most of the reported heterogeneities in tau PET studies, such as primary age‐related tauopathy (PART) and proposed subtypes and heterogeneity in the patterns of tau distribution.^31^, ^50^ Similar methods can be used to select a brain region as reference to scale the tissue ratios. Attempts to define a universal cerebellar tau mask are already underway,^51^ but will require testing with all tau tracers to assess whether it improves the CTR_z_ accuracy.

There are several limitations of the present study. First, similar to the Centiloid method, the mask and scales for some of the tau tracers included here were generated from a limited number of available participant datasets. Second, the masks and scales were generated with elderly CU Aβ− controls and AD Aβ+ patients. A scale generated with young adult controls devoid of cortical tau pathology might hypothetically prove more sensitive to low levels of tau pathology. That said, ongoing studies with ^18^F‐MK6240 and ^18^F‐FTP comparing young adult controls with elderly controls show no significant differences in the tau signal^52^ between young and elderly controls. Third, the performance of the masks and scales were not tested in longitudinal studies and therefore we cannot assess the reproducibility of the method. However, the CenTauR framework is flexible in several key aspects: (1) while the results presented here are the average of left and right hemispheres, data can be expressed unilaterally to characterize potential asymmetries in tau deposition; (2) to capture early cortical tau deposition in the inferior and middle temporal gyri, the MTL CTR_z_ could be subtracted from the meta temporal CTR_z_; (3) similar to what was proposed with the Centiloid method, it allows to resample a CTR_z_ parametric image, either with a different atlas template, using SPM or with a different image analysis pipeline or software, once all voxels are transformed into CTR_z_ parametric images using one of the provided equations (for a global transformation, we suggest using the temporoparietal equation [Figure S13]); and (4) it provides a comprehensive scheme to facilitate and standardize head‐to‐head comparisons between tau tracers.^53^, ^54^ Moreover, and in contrast to the Centiloid approach, by incorporating the tracer‐specific “noise” into the measurement, the CenTauR_z_ approach provides a more robust and meaningful underpinning for head‐to‐head comparisons between these tracers. Last, the modular approach also allows the examination of certain brain regions separately given that they behave differently over time, with for example the MTL accumulating tau early but also plateauing early, or the temporoparietal that seems to be the most sensitive region to capture tau accumulation in the brain, and likely large enough to provide robust statements of changes in tau burden in a clinical trial.^55^, ^56^

In conclusion, we constructed several universal tau PET–specific cortical masks for the AD continuum based on all the commonly used tau tracers, and a universal standard scale, the CenTauR_z_, designed to capture the location and degree of abnormality of tau pathology that can be applied across tracers and across centers. While the CenTauR scheme does not answer *all* questions about measuring tau deposits, it establishes a robust and reproducible standard framework from which to build upon, and to be implemented in the clinic and applied in therapeutic trials.

## CONFLICT OF INTEREST STATEMENT

Victor Villemagne has received research grants from NHMRC (GNT2001320), the Aging Mind Foundation (DAF2255207) and NIH (2P01AG025204‐16) and is and has been a consultant or paid speaker at sponsored conference sessions for Eli Lilly, Life Molecular Imaging, ACE Barcelona, and IXICO. Sandra Sanabria Bohorquez and Robby Weimer are a full‐time employees and stock owners of Roche. Santiago Bullich and Andrew Stephen are full‐time employees of Life Molecular Imaging GmbH. Hitoshi Shimada and Makoto Higuchi hold patents on compounds related to the present report (JP 5422782/EP 12 884 742.3/CA2894994/HK1208672). Christopher C. Rowe has received research grants from NHMRC, Enigma Australia, Biogen, Eisai, and Abbvie. He is on the scientific advisory board for Cerveau Technologies and consulted for Prothena, Eisai, Roche, and Biogen Australia. Oskar Hansson has acquired research support (for the institution) from ADx, AVID Radiopharmaceuticals, Biogen, Eli Lilly, Eisai, Fujirebio, GE Healthcare, Pfizer, and Roche. In the past 2 years, he has received consultancy/speaker fees from AC Immune, Amylyx, Alzpath, BioArctic, Biogen, Cerveau, Eisai, Eli Lilly, Fujirebio, Genentech, Merck, Novartis, Novo Nordisk, Roche, Sanofi, and Siemens. The other authors did not report any conflict of interest.

## CONSENT STATEMENT

All participants gave written consent for publication of de‐identified data.

## COLLABORATORS

The following are the published list of collaborators associated with ADNI:

Michael Weiner, MD (UC San Francisco, Principal Investigator, Executive Committee); Paul Aisen, MD (UC San Diego, ADCS PI and Director of Coordinating Center Clinical Core, Executive Committee, Clinical Core Leaders); Ronald Petersen, MD, PhD (Mayo Clinic, Rochester, Executive Committee, Clinical Core Leader); Clifford R. Jack, Jr., MD (Mayo Clinic, Rochester, Executive Committee, MRI Core Leader); William Jagust, MD (UC Berkeley, Executive Committee; PET Core Leader); John Q. Trojanowki, MD, PhD (U Pennsylvania, Executive Committee, Biomarkers Core Leader); Arthur W. Toga, PhD (USC, Executive Committee, Informatics Core Leader); Laurel Beckett, PhD (UC Davis, Executive Committee, Biostatistics Core Leader); Robert C. Green, MD, MPH (Brigham and Women's Hospital, Harvard Medical School, Executive Committee and Chair of Data and Publication Committee); Andrew J. Saykin, PsyD (Indiana University, Executive Committee, Genetics Core Leader); John Morris, MD (Washington University St. Louis, Executive Committee, Neuropathology Core Leader); Leslie M. Shaw (University of Pennsylvania, Executive Committee, Biomarkers Core Leader); Enchi Liu, PhD (Janssen Alzheimer Immunotherapy, ADNI 2 Private Partner Scientific Board Chair); Tom Montine, MD, PhD (University of Washington); Ronald G. Thomas, PhD (UC San Diego); Michael Donohue, PhD (UC San Diego); Sarah Walter, MSc (UC San Diego); Devon Gessert (UC San Diego); Tamie Sather, MS (UC San Diego); Gus Jiminez, MBS (UC San Diego); Danielle Harvey, PhD (UC Davis); Michael Donohue, PhD (UC San Diego); Matthew Bernstein, PhD (Mayo Clinic, Rochester); Nick Fox, MD (University of London); Paul Thompson, PhD (USC School of Medicine); Norbert Schuff, PhD (UCSF MRI); Charles DeCArli, MD (UC Davis); Bret Borowski, RT (Mayo Clinic); Jeff Gunter, PhD (Mayo Clinic); Matt Senjem, MS (Mayo Clinic); Prashanthi Vemuri, PhD (Mayo Clinic); David Jones, MD (Mayo Clinic); Kejal Kantarci (Mayo Clinic); Chad Ward (Mayo Clinic); Robert A. Koeppe, PhD (University of Michigan, PET Core Leader); Norm Foster, MD (University of Utah); Eric M. Reiman, MD (Banner Alzheimer's Institute); Kewei Chen, PhD (Banner Alzheimer's Institute); Chet Mathis, MD (University of Pittsburgh); Susan Landau, PhD (UC Berkeley); Nigel J. Cairns, PhD, MRCPath (Washington University St. Louis); Erin Householder (Washington University St. Louis); Lisa Taylor Reinwald, BA, HTL (Washington University St. Louis); Virginia Lee, PhD, MBA (UPenn School of Medicine); Magdalena Korecka, PhD (UPenn School of Medicine); Michal Figurski, PhD (UPenn School of Medicine); Karen Crawford (USC); Scott Neu, PhD (USC); Tatiana M. Foroud, PhD (Indiana University); Steven Potkin, MD UC (UC Irvine); Li Shen, PhD (Indiana University); Faber Kelley, MS, CCRC (Indiana University); Sungeun Kim, PhD (Indiana University); Kwangsik Nho, PhD (Indiana University); Zaven Kachaturian, PhD (Khachaturian, Radebaugh & Associates, Inc and Alzheimer's Association's Ronald and Nancy Reagan's Research Institute); Richard Frank, MD, PhD (General Electric); Peter J. Snyder, PhD (Brown University); Susan Molchan, PhD (National Institute on Aging/National Institutes of Health); Jeffrey Kaye, MD (Oregon Health and Science University); Joseph Quinn, MD (Oregon Health and Science University); Betty Lind, BS (Oregon Health and Science University); Raina Carter, BA (Oregon Health and Science University); Sara Dolen, BS (Oregon Health and Science University); Lon S. Schneider, MD (University of Southern California); Sonia Pawluczyk, MD (University of Southern California); Mauricio Beccera, BS (University of Southern California); Liberty Teodoro, RN (University of Southern California); Bryan M. Spann, DO, PhD (University of Southern California); James Brewer, MD, PhD (University of California San Diego); Helen Vanderswag, RN (University of California San Diego); Adam Fleisher, MD (University of California San Diego); Judith L. Heidebrink, MD, MS (University of Michigan); Joanne L. Lord, LPN, BA, CCRC (University of Michigan); Ronald Petersen, MD, PhD (Mayo Clinic, Rochester); Sara S. Mason, RN (Mayo Clinic, Rochester); Colleen S. Albers, RN (Mayo Clinic, Rochester); David Knopman, MD (Mayo Clinic, Rochester); Kris Johnson, RN (Mayo Clinic, Rochester); Rachelle S. Doody, MD, PhD (Baylor College of Medicine); Javier Villanueva Meyer, MD (Baylor College of Medicine); Munir Chowdhury, MBBS, MS (Baylor College of Medicine); Susan Rountree, MD (Baylor College of Medicine); Mimi Dang, MD (Baylor College of Medicine); Yaakov Stern, PhD (Columbia University Medical Center); Lawrence S. Honig, MD, PhD (Columbia University Medical Center); Karen L. Bell, MD (Columbia University Medical Center); Beau Ances, MD (Washington University, St. Louis); John C. Morris, MD (Washington University, St. Louis); Maria Carroll, RN, MSN (Washington University, St. Louis); Sue Leon, RN, MSN (Washington University, St. Louis); Erin Householder, MS, CCRP (Washington University, St. Louis); Mark A. Mintun, MD (Washington University, St. Louis); Stacy Schneider, APRN, BC, GNP (Washington University, St. Louis); Angela Oliver, RN, BSN, MSG (Washington University, St. Louis); Daniel Marson, JD, PhD (University of Alabama Birmingham); Randall Griffith, PhD, ABPP (University of Alabama Birmingham); David Clark, MD (University of Alabama Birmingham); David Geldmacher, MD (University of Alabama Birmingham); John Brockington, MD (University of Alabama Birmingham); Erik Roberson, MD (University of Alabama Birmingham); Hillel Grossman, MD (Mount Sinai School of Medicine); Effie Mitsis, PhD (Mount Sinai School of Medicine); Leyla deToledo‐Morrell, PhD (Rush University Medical Center); Raj C. Shah, MD (Rush University Medical Center); Ranjan Duara, MD (Wien Center); Daniel Varon, MD (Wien Center); Maria T. Greig, HP (Wien Center); Peggy Roberts, CNA (Wien Center); Marilyn Albert, PhD (Johns Hopkins University); Chiadi Onyike, MD (Johns Hopkins University); Daniel D'Agostino II, BS (Johns Hopkins University); Stephanie Kielb, BS (Johns Hopkins University); James E. Galvin, MD, MPH (New York University); Dana M. Pogorelec (New York University); Brittany Cerbone (New York University); Christina A. Michel (New York University); Henry Rusinek, PhD (New York University); Mony J. de Leon, EdD (New York University); Lidia Glodzik, MD, PhD (New York University); Susan De Santi, PhD (New York University); P. Murali Doraiswamy, MD (Duke University Medical Center); Jeffrey R. Petrella, MD (Duke University Medical Center); Terence Z. Wong, MD (Duke University Medical Center); Steven E. Arnold, MD (University of Pennsylvania); Jason H. Karlawish, MD (University of Pennsylvania); David Wolk, MD (University of Pennsylvania); Charles D. Smith, MD (University of Kentucky); Greg Jicha, MD (University of Kentucky); Peter Hardy, PhD (University of Kentucky); Partha Sinha, PhD (University of Kentucky); Elizabeth Oates, MD (University of Kentucky); Gary Conrad, MD (University of Kentucky); Oscar L. Lopez, MD (University of Pittsburgh); MaryAnn Oakley, MA (University of Pittsburgh); Donna M. Simpson, CRNP, MPH (University of Pittsburgh); Anton P. Porsteinsson, MD (University of Rochester Medical Center); Bonnie S. Goldstein, MS, NP (University of Rochester Medical Center); Kim Martin, RN (University of Rochester Medical Center); Kelly M. Makino, BS (University of Rochester Medical Center); M. Saleem Ismail, MD (University of Rochester Medical Center); Connie Brand, RN (University of Rochester Medical Center); Ruth A. Mulnard, DNSc, RN, FAAN (University of California, Irvine); Gaby Thai, MD (University of California, Irvine); Catherine Mc Adams Ortiz, MSN, RN, A/GNP (University of California, Irvine); Kyle Womack, MD (University of Texas Southwestern Medical School); Dana Mathews, MD, PhD (University of Texas Southwestern Medical School); Mary Quiceno, MD (University of Texas Southwestern Medical School); Ramon Diaz Arrastia, MD, PhD (University of Texas Southwestern Medical School); Richard King, MD (University of Texas Southwestern Medical School); Myron Weiner, MD (University of Texas Southwestern Medical School); Kristen Martin Cook, MA (University of Texas Southwestern Medical School); Michael DeVous, PhD (University of Texas Southwestern Medical School); Allan I. Levey, MD, PhD (Emory University); James J. Lah, MD, PhD (Emory University); Janet S. Cellar, DNP, PMHCNS BC (Emory University); Jeffrey M. Burns, MD (University of Kansas, Medical Center); Heather S. Anderson, MD (University of Kansas, Medical Center); Russell H. Swerdlow, MD (University of Kansas, Medical Center); Liana Apostolova, MD (University of California, Los Angeles); Kathleen Tingus, PhD (University of California, Los Angeles); Ellen Woo, PhD (University of California, Los Angeles); Daniel H.S. Silverman, MD, PhD (University of California, Los Angeles); Po H. Lu, PsyD (University of California, Los Angeles); George Bartzokis, MD (University of California, Los Angeles); Neill R. Graff Radford, MBBCH, FRCP (London) (Mayo Clinic, Jacksonville); Francine Parfitt, MSH, CCRC (Mayo Clinic, Jacksonville); Tracy Kendall, BA, CCRP (Mayo Clinic, Jacksonville); Heather Johnson, MLS, CCRP (Mayo Clinic, Jacksonville); Martin R. Farlow, MD (Indiana University); Ann Marie Hake, MD (Indiana University); Brandy R. Matthews, MD (Indiana University); Scott Herring, RN, CCRC (Indiana University); Cynthia Hunt, BS, CCRP (Indiana University); Christopher H. van Dyck, MD (Yale University School of Medicine); Richard E. Carson, PhD (Yale University School of Medicine); Martha G. MacAvoy, PhD (Yale University School of Medicine); Howard Chertkow, MD (McGill Univ., Montreal Jewish General Hospital); Howard Bergman, MD (McGill Univ., Montreal Jewish General Hospital); Chris Hosein, Med (McGill Univ., Montreal Jewish General Hospital); Sandra Black, MD, FRCPC (Sunnybrook Health Sciences, Ontario); Dr Bojana Stefanovic (Sunnybrook Health Sciences, Ontario); Curtis Caldwell, PhD (Sunnybrook Health Sciences, Ontario); Ging Yuek Robin Hsiung, MD, MHSc, FRCPC (U.B.C. Clinic for AD & Related Disorders); Howard Feldman, MD, FRCPC (U.B.C. Clinic for AD & Related Disorders); Benita Mudge, BS (U.B.C. Clinic for AD & Related Disorders); Michele Assaly, MA Past (U.B.C. Clinic for AD & Related Disorders); Andrew Kertesz, MD (Cognitive Neurology St. Joseph's, Ontario); John Rogers, MD (Cognitive Neurology St. Joseph's, Ontario); Dick Trost, PhD (Cognitive Neurology St. Joseph's, Ontario); Charles Bernick, MD (Cleveland Clinic Lou Ruvo Center for Brain Health); Donna Munic, PhD (Cleveland Clinic Lou Ruvo Center for Brain Health); Diana Kerwin, MD (Northwestern University); Marek Marsel Mesulam, MD (Northwestern University); Kristine Lipowski, BA (Northwestern University); Chuang Kuo Wu, MD, PhD (Northwestern University); Nancy Johnson, PhD (Northwestern University); Carl Sadowsky, MD (Premiere Research Inst [Palm Beach Neurology]); Walter Martinez, MD (Premiere Research Inst [Palm Beach Neurology]); Teresa Villena, MD (Premiere Research Inst [Palm Beach Neurology]); Raymond Scott Turner, MD, PhD (Georgetown University Medical Center); Kathleen Johnson, NP (Georgetown University Medical Center); Brigid Reynolds, NP (Georgetown University Medical Center); Reisa A. Sperling, MD (Brigham and Women's Hospital); Keith A. Johnson, MD (Brigham and Women's Hospital); Gad Marshall, MD (Brigham and Women's Hospital); Meghan Frey (Brigham and Women's Hospital); Jerome Yesavage, MD (Stanford University); Joy L. Taylor, PhD (Stanford University); Barton Lane, MD (Stanford University); Allyson Rosen, PhD (Stanford University); Jared Tinklenberg, MD (Stanford University); Marwan N. Sabbagh, MD (Banner Sun Health Research Institute); Christine M. Belden, PsyD (Banner Sun Health Research Institute); Sandra A. Jacobson, MD (Banner Sun Health Research Institute); Sherye A. Sirrel, MS (Banner Sun Health Research Institute); Neil Kowall, MD (Boston University); Ronald Killiany, PhD (Boston University); Andrew E. Budson, MD (Boston University); Alexander Norbash, MD (Boston University); Patricia Lynn Johnson, BA (Boston University); Thomas O. Obisesan, MD, MPH (Howard University); Saba Wolday, MSc (Howard University); Joanne Allard, PhD (Howard University); Alan Lerner, MD (Case Western Reserve University); Paula Ogrocki, PhD (Case Western Reserve University); Leon Hudson, MPH (Case Western Reserve University); Evan Fletcher, PhD (University of California, Davis Sacramento); Owen Carmichael, PhD (University of California, Davis Sacramento); John Olichney, MD (University of California, Davis Sacramento); Charles DeCarli, MD (University of California, Davis Sacramento); Smita Kittur, MD (Neurological Care of CNY); Michael Borrie, MB ChB (Parkwood Hospital); T. Y. Lee, PhD (Parkwood Hospital); Dr Rob Bartha, PhD (Parkwood Hospital); Sterling Johnson, PhD (University of Wisconsin); Sanjay Asthana, MD (University of Wisconsin); Cynthia M. Carlsson, MD (University of Wisconsin); Steven G. Potkin, MD (University of California, Irvine BIC); Adrian Preda, MD (University of California, Irvine BIC); Dana Nguyen, PhD (University of California, Irvine BIC); Pierre Tariot, MD (Banner Alzheimer's Institute); Adam Fleisher, MD (Banner Alzheimer's Institute); Stephanie Reeder, BA (Banner Alzheimer's Institute); Vernice Bates, MD (Dent Neurologic Institute); Horacio Capote, MD (Dent Neurologic Institute); Michelle Rainka, PharmD, CCRP (Dent Neurologic Institute); Douglas W. Scharre, MD (Ohio State University); Maria Kataki, MD, PhD (Ohio State University); Anahita Adeli, MD (Ohio State University); Earl A. Zimmerman, MD (Albany Medical College); Dzintra Celmins, MD (Albany Medical College); Alice D. Brown, FNP (Albany Medical College); Godfrey D. Pearlson, MD (Hartford Hosp, Olin Neuropsychiatry Research Center); Karen Blank, MD (Hartford Hosp, Olin Neuropsychiatry Research Center); Karen Anderson, RN (Hartford Hosp, Olin Neuropsychiatry Research Center); Robert B. Santulli, MD (Dartmouth Hitchcock Medical Center); Tamar J. Kitzmiller (Dartmouth Hitchcock Medical Center); Eben S. Schwartz, PhD (Dartmouth Hitchcock Medical Center); Kaycee M. Sink, MD, MAS (Wake Forest University Health Sciences); Jeff D. Williamson, MD, MHS (Wake Forest University Health Sciences); Pradeep Garg, PhD (Wake Forest University Health Sciences); Franklin Watkins, MD (Wake Forest University Health Sciences); Brian R. Ott, MD (Rhode Island Hospital); Henry Querfurth, MD (Rhode Island Hospital); Geoffrey Tremont, PhD (Rhode Island Hospital); Stephen Salloway, MD, MS (Butler Hospital); Paul Malloy, PhD (Butler Hospital); Stephen Correia, PhD (Butler Hospital); Howard J. Rosen, MD (UC San Francisco); Bruce L. Miller, MD (UC San Francisco); Jacobo Mintzer, MD, MBA (Medical University South Carolina); Kenneth Spicer, MD, PhD (Medical University South Carolina); David Bachman, MD (Medical University South Carolina); Elizabeth Finger, MD (St. Joseph's Health Care); Stephen Pasternak, MD (St. Joseph's Health Care); Irina Rachinsky, MD (St. Joseph's Health Care); John Rogers, MD (St. Joseph's Health Care); Andrew Kertesz, MD (St. Joseph's Health Care); Dick Drost, MD (St. Joseph's Health Care); Nunzio Pomara, MD (Nathan Kline Institute); Raymundo Hernando, MD (Nathan Kline Institute); Antero Sarrael, MD (Nathan Kline Institute); Susan K. Schultz, MD (University of Iowa College of Medicine, Iowa City); Laura L. Boles Ponto, PhD (University of Iowa College of Medicine, Iowa City); Hyungsub Shim, MD (University of Iowa College of Medicine, Iowa City); Karen Elizabeth Smith, RN (University of Iowa College of Medicine, Iowa City); Norman Relkin, MD, PhD (Cornell University); Gloria Chaing, MD (Cornell University); Lisa Raudin, PhD (Cornell University); Amanda Smith, MD (University of South Florida: USF Health Byrd Alzheimer's Institute); Kristin Fargher, MD (University of South Florida: USF Health Byrd Alzheimer's Institute); Balebail Ashok Raj, MD (University of South Florida: USF Health Byrd Alzheimer's Institute)

AIBL's large, multidisciplinary research team includes the following active members.

Arti Appannah, Mary Barnes, Kevin Barnham, Justin Bedo, Shayne Bellingham, Lynette Bon, Pierrick Bourgeat, Belinda Brown, Rachel Buckley, Samantha Burnham, Ashley Bush, Graeme Chandler, Karren Chen, Roger Clarnette, Steven Collins, Ian Cooke, Tiffany Cowie, Kay Cox, Emily Cuningham, Elizabeth Cyarto, Phuong Anh Vu Dang, David Darby, Patricia Desmond, James Doecke, Vincent Dore, Harriet Downing, Belinda Dridan, Konsta Duesing, Michael Fahey, Maree Farrow, Noel Faux, Michael Fenech, Shane Fernandez, Binosha Fernando, Chris Fowler, Maxime Francois, Jurgen Fripp, Shaun Frost, Samantha Gardener, Simon Gibson, Petra Graham, Veer Gupta, David Hansen, Karra Harrington, Andy Hill, Eugene Hone, Maryam Hor, Malcolm Horne, Brenda Huckstepp, Andrew Jones, Gareth Jones, Adrian Kamer, Yogi Kanagasingam, Lisa Keam, Adam Kowalczyk, Betty Krivdic, Chiou Peng Lam, Fiona Lamb, Nicola Lautenschlager, Simon Laws, Wayne Leifert, Nat Lenzo, Hugo Leroux, Falak Lftikhar, Qiao‐Xin Li, Florence Lim, Lucy Lim, Linda Lockett, Kathy Lucas, Mark Mano, Caroline Marczak, Georgia Martins, Paul Maruff, Yumiko Matsumoto, Sabine Bird,Rachel McKay, Rachel Mulligan, Tabitha Nash, Julie Nigro, Graeme O'Keefe, Kevin Ong, Bernadette Parker, Steve Pedrini, Jeremiah Peiffer, Sveltana Pejoska, Lisa Penny, Keyla Perez, Kelly Pertile, Pramit Phal, Tenielle Porter, Stephanie Rainey‐Smith, Parnesh Raniga, Alan Rembach, Carolina Restrepo, Malcolm Riley, Blaine Roberts, Jo Robertson, Mark Rodrigues, Alicia Rooney, Rebecca Rumble, Tim Ryan, Mather Samuel, Ian Saunders, Greg Savage, Brendan Silbert, Hamid Sohrabi, Julie Syrette, Cassandra Szoeke, Kevin Taddei, Tania Taddei, Sherilyn Tan, Michelle Tegg, Philip Thomas, Darshan Trivedi, Brett Trounson, Robyn Veljanovski, Giuseppe Verdile, Victor Villemagne, Irene Volitakis, Cassandra Vockler, Michael Vovos, Freda Vrantsidis, Stacey Walker, Andrew Watt, Mike Weinborn, Bill Wilson, Michael Woodward, Olga Yastrubetskaya, Paul Yates, Ping Zhang.

## Supporting information

Supporting Information

Supporting Information
